# An infected aneurysm of the vertebral artery following cervical pyogenic spondylitis: a case report and literature review

**DOI:** 10.1186/s12891-020-03881-3

**Published:** 2021-01-06

**Authors:** Takahiro Furukawa, Keisuke Masuda, Hideki Shigematsu, Masato Tanaka, Akinori Okuda, Sachiko Kawasaki, Yuma Suga, Yusuke Yamamoto, Yasuhito Tanaka

**Affiliations:** 1 Department of Orthopedic Surgery, Nara Medical University, 840 Shijo-cho, Kashihara City, 6348522 Nara Japan; 2 Department of Emergency and Critical Care Medicine, Nara Medical University, 840 Shijo-cho, Kashihara City, 6348522 Nara Japan

**Keywords:** Pyogenic spondylitis, Infected aneurysm, Vertebral artery, Ultrasonographic examination, Case report

## Abstract

**Background:**

An important complication of pyogenic spondylitis is aneurysms in the adjacent arteries. There are reports of abdominal aortic or iliac aneurysms, but there are few reports describing infected aneurysms of the vertebral artery. Furthermore, there are no reports describing infected aneurysms of the vertebral arteries following cervical pyogenic spondylitis. We report a rare case of an infected aneurysm of the vertebral artery as a complication of cervical pyogenic spondylitis, which was successfully treated by endovascular treatment.

**Case presentation:**

Cervical magnetic resonance imaging (MRI) of a 59-year-old man who complained of severe neck pain showed pyogenic spondylitis. Although he was treated extensively by antibiotic therapy, his neck pain did not improve. Follow-up MRI showed the presence of a cyst, which was initially considered an abscess, and therefore, treatment initially included guided tapping and suction under ultrasonography. However, under ultrasonographic examination an aneurysm was detected. The contrast-enhanced computed tomography (CT) scan showed an aneurysm of the vertebral artery. Following endovascular treatment (parent artery occlusion: PAO), the patient’s neck pain disappeared completely.

**Conclusion:**

Although there are several reports of infected aneurysms of the vertebral arteries, this is the first report describing an infected aneurysm of the vertebral artery as a result of cervical pyogenic spondylitis.

Whenever a paraspinal cyst exist at the site of infection, we recommend that clinicians use not only X-ray, conventional CT, and MRI to examine the cyst, but ultrasonography and contrast-enhanced CT as well because of the possibility of an aneurysms in neighboring blood vessels. It is necessary to evaluate the morphology of the aneurysm to determine the treatment required.

## Background

The number of patients being hospitalized because of pyogenic spondylitis is increasing annually [1]. Abscesses may result from pyogenic spondylitis. Although an aneurysm at an adjacent artery is one of the important complications associated with pyogenic spondylitis, aneurysms might be misdiagnosed as abscesses. There have been reports of abdominal aortic or iliac aneurysms following pyogenic spondylitis [2–6]; however, no reports describing infected aneurysms of the vertebral arteries following cervical pyogenic spondylitis exist. We report a rare case of an infected aneurysm of the vertebral artery following cervical pyogenic spondylitis, which was successfully managed with endovascular treatment.

## Case presentation

**Fig. 1:**
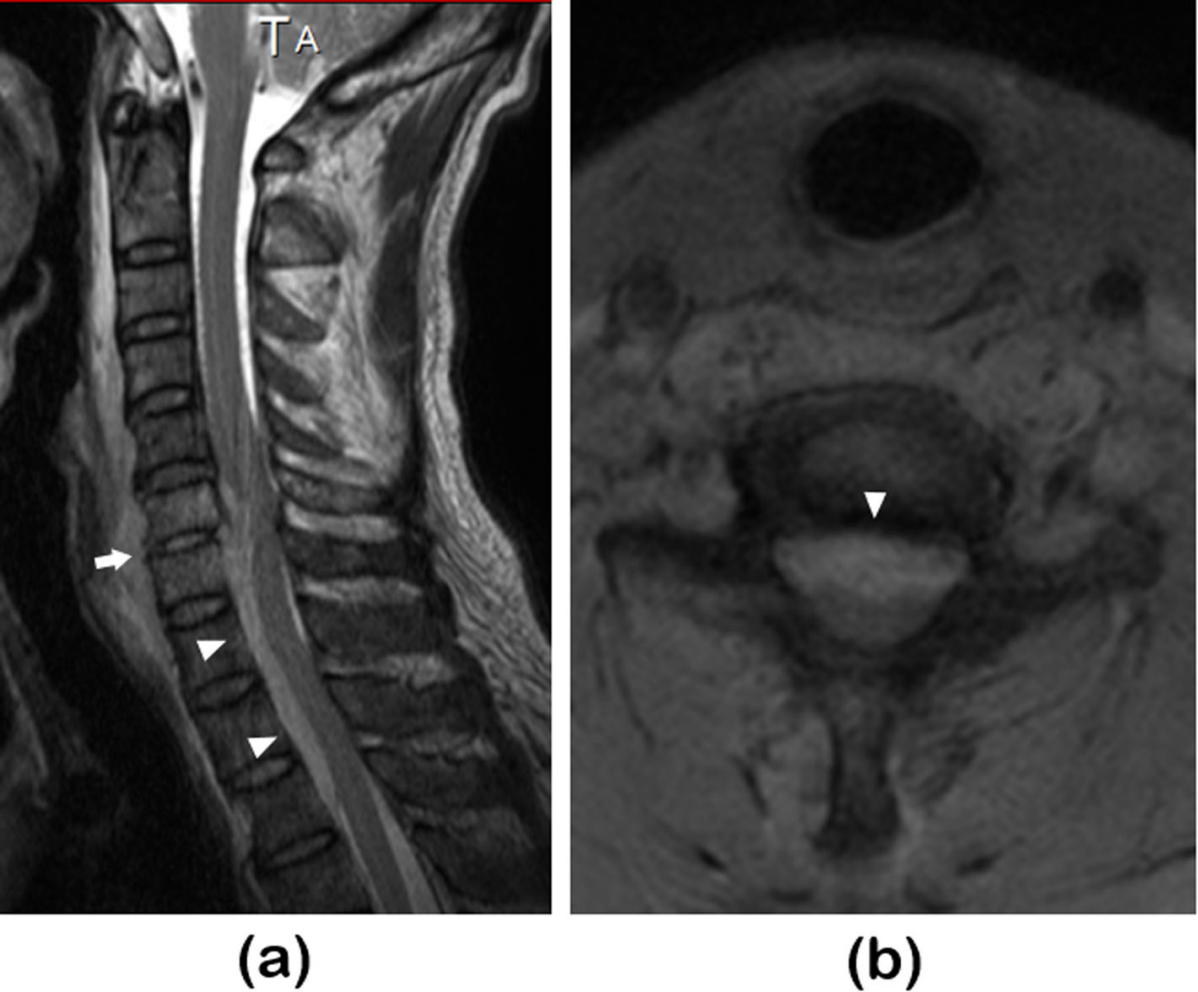
Magnetic resonance imaging performed on initial hospitalization at the referring hospital. **a** T2-weighted sagittal image revealed a high intensity lesion at the C6/C7 vertebral body (arrow), retropharyngeal space, and epidural space (arrowhead). The epidural lesion had compressed the dural sac from the ventral side **b** T2-weighted axial image at C6/7 revealed a high intensity lesion at the epidural space (arrowhead) that had compressed the dural sac from the ventral side

A 59-year-old man with a medical history of dyslipidemia was suffering from continuous neck pain. Three days after the appearance of neck pain, he visited a nearby clinic and was treated conservatively using analgesics only. Two weeks later, he was referred to another hospital because of a high fever of 38.6° C and increased inflammatory markers on a blood examination [C-reactive protein (CRP) was 31.55 mg/dl and white blood cell (WBC) count was 31,000/µl]. He was hospitalized on the same day (day 0) because magnetic resonance imaging (MRI) showed cervical pyogenic spondylitis and an extradural abscess at C6 and C7 (Fig. 1).

**Fig. 2:**
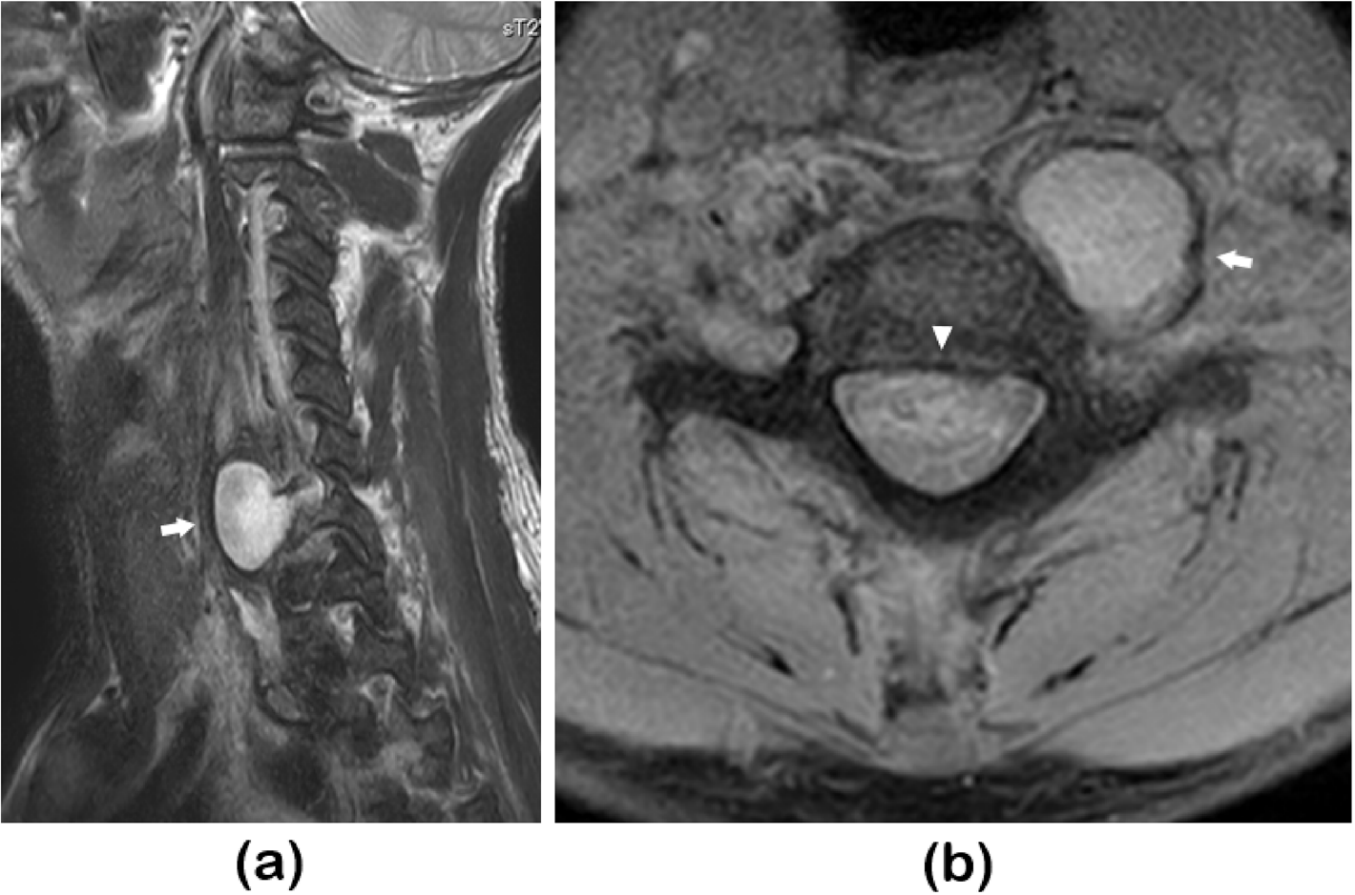
Follow-up magnetic resonance imaging performed at referring hospital **a** T2 weighted parasagittal image at left side revealed a cyst at C6/C7 level (arrow) **b** T2-weighted axial image revealed a cyst at the retropharyngeal space, adjacent to the infected vertebral body (arrow). Dural sac compression by the epidural abscess remained (arrowhead)

He was treated with a combination of antibiotics including vancomycin and cefazolin and provided with a cervical collar. *Staphylococcus aureus* was detected in the blood culture on day 5, and cefazolin was subsequently administered. The inflammation improved and tests performed on day 17 showed CRP of 3.86 mg/dl and WBC count of 5700/µl. However, the patient reported that their neck pain had worsened and follow-up MRI on day 17 indicated the presence of a cyst at the retropharyngeal space adjacent to the infected vertebral body (Fig. 2). The patient was then transferred to our hospital for further treatment on day 20.

Once he had been transferred, his body temperature was recorded at 37.7 ° C and the rest of his vital signs were normal. CRP was 5.03 mg/dl and WBC count was 7400/µl. He felt severe neck pain with a visual analogue scale (VAS) score of 8.0. Physical examination revealed muscle weakness at the bilateral finger extension. There was no evidence of pyramidal signs or sensory disturbance.

**Fig. 3:**
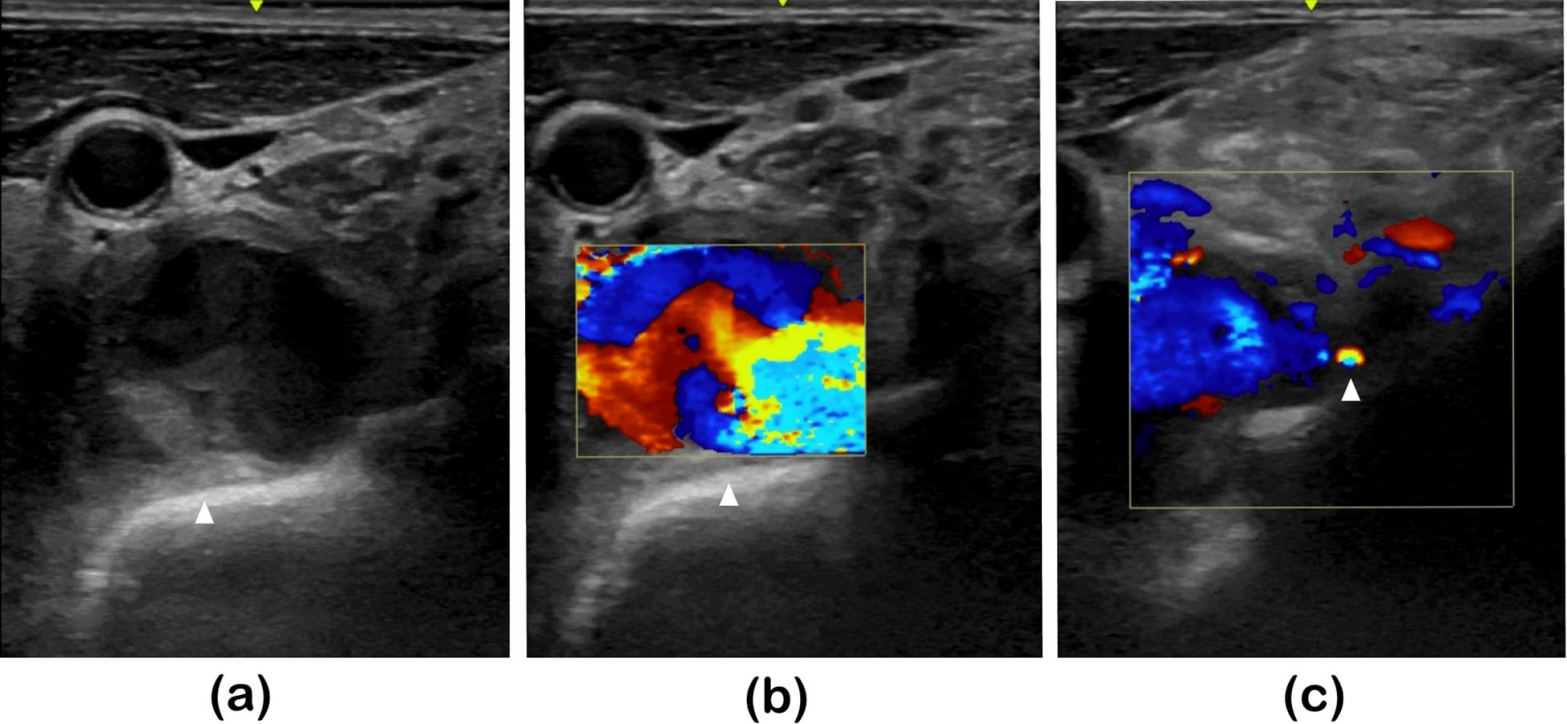
Ultrasonographic examination of the neck **a** Imaging with normal parameters showed pulsatile turbulence in the cyst (arrowhead) **b** Color Doppler imaging showed heterogeneous blood flow inside the cyst (arrowhead) **c** Color Doppler imaging showed the vertebral artery (arrowhead) linked to the cyst

**Fig. 4:**
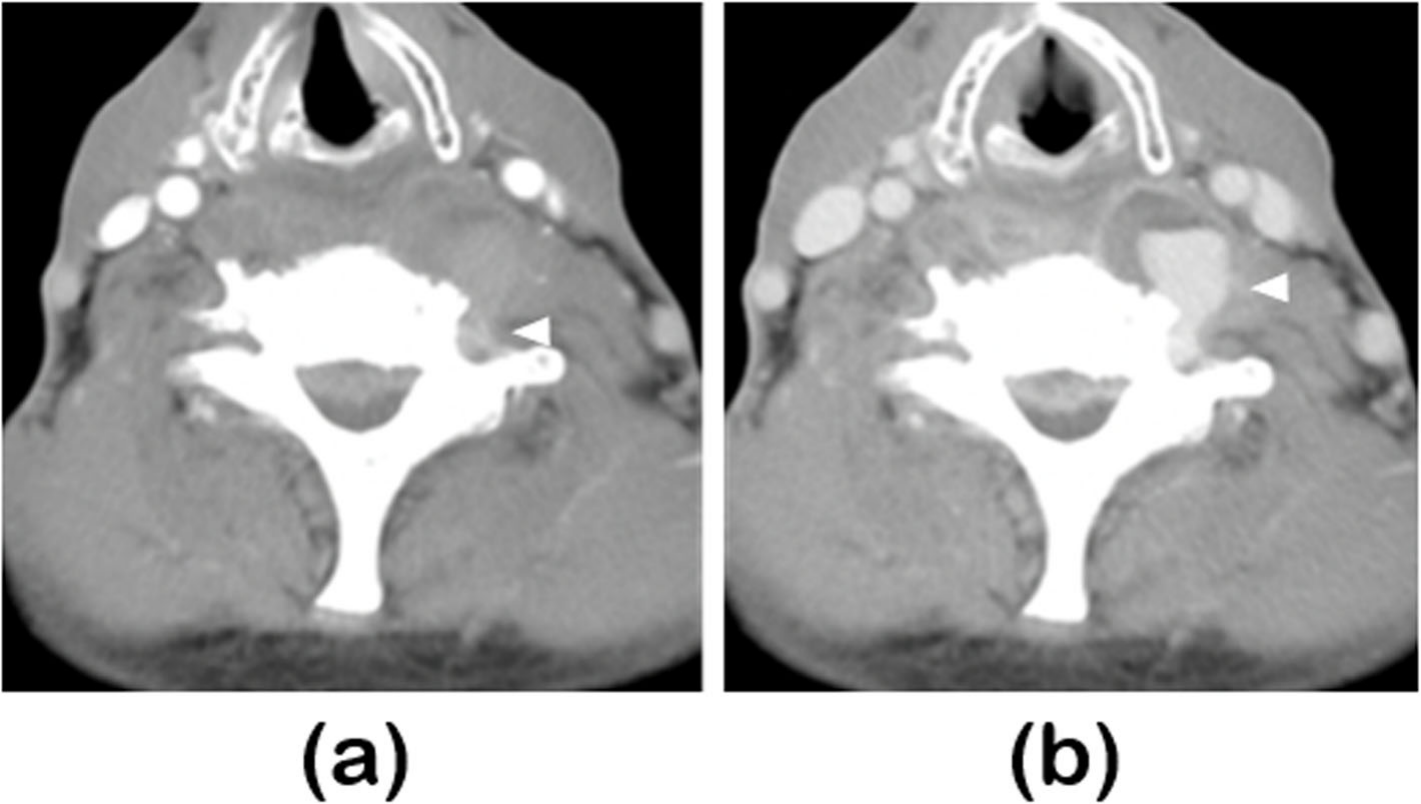
Contrast-enhanced computed tomography. **a** The arterial phase showed slight contrast-enhancement of the left vertebral artery (arrowhead) **b** The delayed phase showed a contrast-enhanced lesion in the cyst (arrowhead). The lesion was linked to the left vertebral artery

**Fig. 5:**
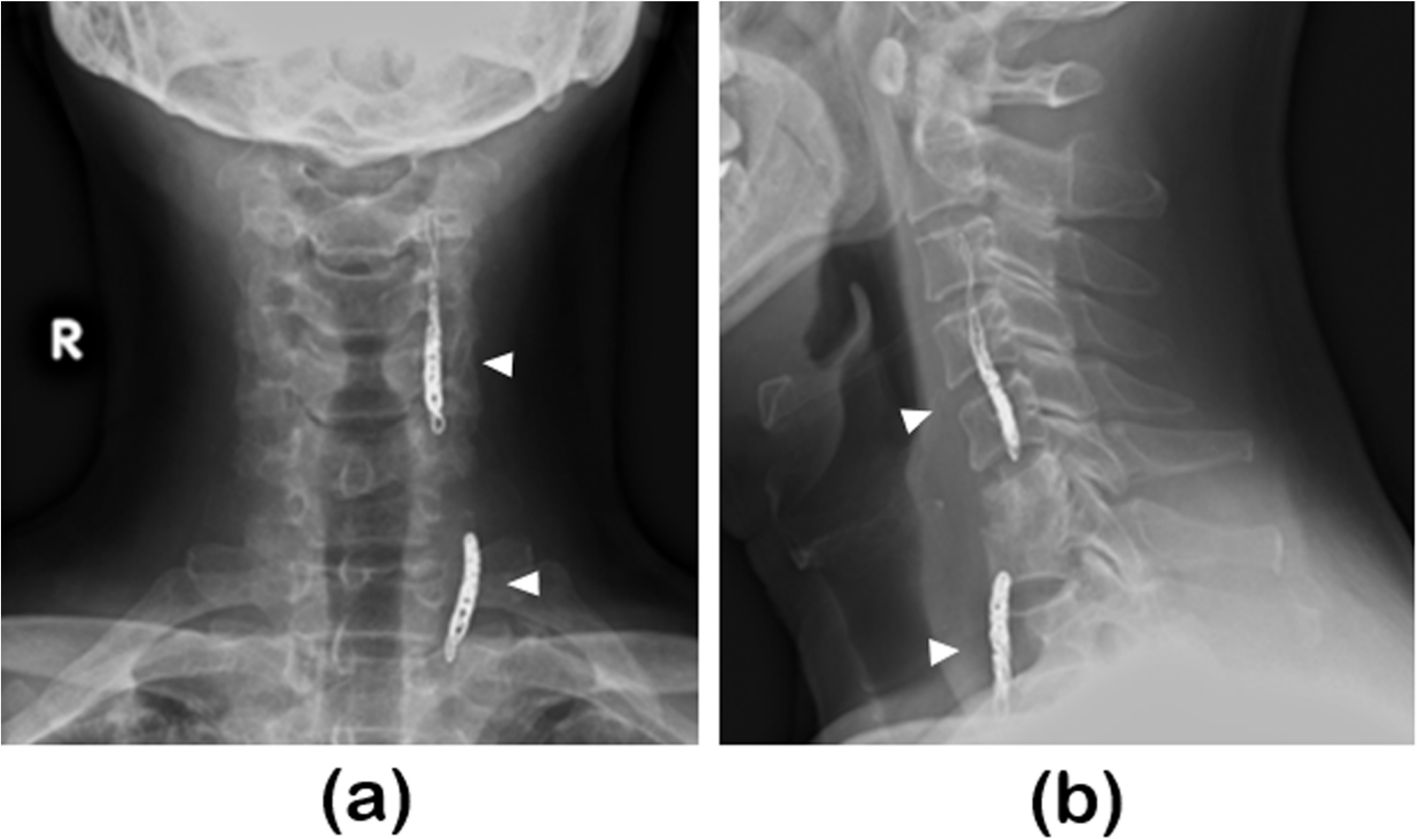
X-rays obtained after endovascular treatment Both the anteroposterior (**a**) and mediolateral views (**b**) showed the coiled left vertebral artery (arrowhead)

An ultrasonographic examination of the neck was performed in order to evaluate the properties of the neck cyst (which was thought to be an abscess). Ultrasonographic examination showed pulsatile turbulence in the cyst (Fig. 3a). Color Doppler ultrasonography showed blood flow inside the cyst, which was linked to the vertebral artery (Fig. 3b and c). Therefore, we suspected the cyst to be a vertebral aneurysm and performed contrast-enhanced computed tomography (CT) for confirmation. The contrast-enhanced CT scans showed blood flow to the cyst from the vertebral artery; therefore, the cyst, initially thought to be an abscess, was diagnosed as an infected aneurysm of the vertebral artery (Fig. 4). The patient underwent endovascular treatment (parent artery occlusion; PAO) at our department of Neurosurgery (Fig. 5) and his neck pain subsequently disappeared completely (VAS was 0).

He was transferred back to the referring hospital on day 47 without complications resulting from the treatment. Cefazolin was administered to the patient until day 58, after which cefaclor was orally administered until day 91 (as an outpatient). CRP was 0.02 mg/dl and WBC count was 4600/µl in a blood examination performed on day 91. When the antibiotic therapy was finished, his muscle weakness recovered and his VAS for neck pain remained 0. The C5-C6 vertebral body was completely fused. At the 2-year follow-up, there were no new lesions or recurrence of the pyogenic spondylitis or aneurysm.

## Discussion

Most incidences of pyogenic spondylitis are reported as bloodstream infections that often infect intervertebral discs [7]. Our case did not result from iatrogenic conditions or an underlying disease, thus, the route of infection was unknown. For pyogenic spondylitis, a biopsy is recommended to determine the responsible bacteria [8]. Initially, biopsy and drainage were recommended because of the presence of a cyst in our case, which thought to be an abscess. Generally, CT-guided or fluoroscopy-guided biopsy is performed for pyogenic spondylitis [9]. In our case, we planned ultrasound-guided biopsy because the cyst and vessels were not deeply located and could be easily detected by ultrasonography in real time. Ultrasonographic findings revealed that the cyst (initially thought to be an abscess) was actually an aneurysm of the vertebral artery.

Although there are reports of infected aneurysms, a limited number of reports have described infected aneurysms of the vertebral arteries as indicated in Table 1 [10–13]. Moreover, to our knowledge, this is the first report describing an infected aneurysm of the vertebral artery due to cervical pyogenic spondylitis. The first infected aneurysm was reported by Osler [14], which had been caused by invasion of the arterial wall by bacteria and its destruction by neutrophils.

**Table 1:** Past reports of infected aneurysms of the vertebral arteries: a systematic review

Reference	Age (years), sex	Cause	Responsible bacteria	Treatment
Flye et al. 1971 [10]	42, Male	Iatrogenic	*S. aureus*	Surgery (rupture)
Singh et al. 2005 [11]	7, Male	Infective endocarditis	Unknown	Surgery
Gupta et al. 2014 [12]	25, Male	Lemierre’s disease	Methicillin-resistant *S. aureus* (MRSA)	Antibiotics
Hashimoto et al. 2015 [13]	75, Male	Cholangitis	*S. aureus*	Endovascular Surgery
Current report	59, Male	Pyogenic spondylitis	*S. aureus*	Endovascular Surgery

*S. aureus: Staphylococcus aureus*

Infected aneurysms are broadly classified into those arising from infection of arterial walls and the infection of existing aneurysms. The routes of infections of arterial walls are further classified into four categories: (1) infected endocarditis [14], (2) adjacent infected lesions [15], (3) bloodstream infection due to a damaged vascular intima [16], (4) infection due to trauma [17]. The route of infection in our case was considered to be the adjacent infected lesion.

In previous reports, the diagnosis of an infected aneurysm of the vertebral artery was confirmed by conventional and contrast-enhanced CT. An aneurysm may be diagnosed by palpation, but this was difficult in our case because the common carotid artery was in close proximity to the aneurysm. Therefore, we confirmed that the cyst (which was initially thought to be an abscess) was an aneurysm by ultrasonographic examination, which is a non-invasive and convenient procedure. Furthermore, contrast-enhanced CT scans showed that the cyst was an aneurysm of the vertebral artery. MRI is generally considered the most sensitive method for examination of pyogenic spondylitis [18]. In our case, during the follow-up consultation, monitoring of the pyogenic spondylitis was performed by MRI and X-ray photography. Since the spine is adjacent to blood vessels, it is difficult to distinguish whether a paraspinal cyst is an abscess or aneurysm. Therefore, it is necessary to exclude aneurysms by ultrasonographic examination or contrast-enhanced CT when a paraspinal cyst is observed, even if the cyst is thought to be an abscess.

The treatment for infected aneurysms of the vertebral arteries, includes surgical treatments for ruptured cases and large aneurysms [10, 11]. In addition, endovascular treatments have been reported for saccular aneurysms [13], and conservative treatment has been reported for asymptomatic fusiform aneurysms [12]. According to the literature, surgery is recommended for ruptured cases and large aneurysms of vertebral artery. When deciding on the management strategy of unruptured aneurysms of the vertebral artery, adopting a treatment strategy similar to that of cerebral aneurysms is desirable. Conservative treatment is indicated for aneurysms with a diameter ≤ 5 mm or ≤ 7 mm, while surgery is recommended for larger aneurysms [19, 20]. Infected aneurysms are particularly more likely to expand or rupture and require aggressive surgery [21]. In terms of aneurysm morphology, it has been reported that saccular aneurysms have a higher risk of rupture than fusiform aneurysms [22], and high-risk saccular aneurysms can be detected by determining their aspect ratio [23, 24]. In addition, if clinical symptoms suggestive of imminent rupture are present, such as pain, or if the diameter of the aneurysm increases rapidly, urgent surgery is required. In summary, we believe that safe, conservative treatment of infected aneurysms of vertebral artery are only possible for small, asymptomatic, fusiform aneurysms. Aneurysms with a diameter of 5 mm or less are particularly good candidates for conservative treatment. Even when electing conservative treatment, it is necessary to perform CT or ultrasonographic follow-up with strict antihypertensive control and be aware of whether the aneurysm diameter has expanded. Before surgery, it is necessary to check the dominant side of the vertebral artery and determine the presence of anomalies such as posterior inferior cerebellar artery (PICA) termination, which is reportedly found in 0.04–1.3% of cases [25–27]. If there is a risk of cerebral infarction due to embolization of the vertebral artery on the side of the aneurysm, occipital artery-PICA bypass or endovascular treatment with a stent should be performed. Since endovascular modalities are developing rapidly, even ruptured aneurysms and aneurysms that require revascularization may be managed endovascularly, except in cases where endovascular treatment is technically difficult or when there are concerns about placing a prosthetic device at the infected site. In the current case, the patient suffered from severe neck pain, and we suspected that rupture of a saccular aneurysm was impending. Minimally invasive endovascular treatment was thus performed. Generally, PAO or stent implantation are performed as the endovascular treatment. We selected PAO because of the sufficient blood flow from the contralateral vertebral artery and concerns about placing an artificial object at the infected site. As a result, cervical pain disappeared and a good outcome was achieved.

In conclusion, we described the treatment of an infected aneurysm of the vertebral artery following cervical pyogenic spondylitis. This report highlights the need to evaluate paraspinal cysts located around the vertebral artery using not only X-ray, conventional CT, and MRI, but also ultrasonographic examination and contrast-enhanced CT considering the possibility of an aneurysm being present. Moreover, we recommend that the morphology of the aneurysm be evaluated to determine the most effective treatment required. The cervical pain of the patient in the current study disappeared after endovascular treatment.

## Data Availability

Not applicable.

## Abbreviations

CRP: C-reactive protein
CT: Computed tomography
MRI: Magnetic resonance imaging
MRSA: Methicillin-resistant *Staphylococcus aureus*
PAO: Parent artery occlusion
PICA: Posterior inferior cerebellar artery
VAS: Visual analogue scale
WBC: White blood cell
